# Connectivity of the Primate Superior Colliculus Mapped by Concurrent Microstimulation and Event-Related fMRI

**DOI:** 10.1371/journal.pone.0003928

**Published:** 2008-12-11

**Authors:** Courtney B. Field, Kevin Johnston, Joseph S. Gati, Ravi S. Menon, Stefan Everling

**Affiliations:** 1 Department of Physiology and Pharmacology, University of Western Ontario, London, Ontario, Canada; 2 Robarts Research Institute, London, Ontario, Canada; University of California Davis, United States of America

## Abstract

**Background:**

Neuroanatomical studies investigating the connectivity of brain areas have heretofore employed procedures in which chemical or viral tracers are injected into an area of interest, and connected areas are subsequently identified using histological techniques. Such experiments require the sacrifice of the animals and do not allow for subsequent electrophysiological studies in the same subjects, rendering a direct investigation of the functional properties of anatomically identified areas impossible.

**Methodology/Principal Findings:**

Here, we used a combination of microstimulation and fMRI in an anesthetized monkey preparation to study the connectivity of the superior colliculus (SC). Microstimulation of the SC resulted in changes in the blood oxygenation level-dependent (BOLD) signals in the SC and in several cortical and subcortical areas consistent with the known connectivity of the SC in primates.

**Conclusions/Significance:**

These findings demonstrates that the concurrent use of microstimulation and fMRI can be used to identify brain networks for further electrophysiological or fMRI investigation.

## Introduction

An essential prerequisite for interpreting neural activity in various areas of the brain is a knowledge of the functional connections of the area of interest. Anterograde and retrograde tract-tracing techniques have proven to be very successful in mapping the connectivity of brain regions in many species, including nonhuman primates [1]–[3]. These techniques require the injection of a substance into a small region of the brain that is then transported up or down the axons [4], [5]. After a waiting period of several days to allow for transport of the tracer substance, the animal is sacrificed, and the tissue is then processed and microscopically analyzed. A popular tracer has been the enzyme horseradish peroxidase [6]. More recently, viral tracers such as rabies virus or herpes simplex have been used successfully. These tracers have the added advantage of allowing tracing of transsynaptic connections [7], [8]. These anatomical techniques have yielded considerable insight into the neural organization of many systems in a variety of species. In addition, application of these techniques has demonstrated that the exact location of many brain areas differs to some degree from individual to individual (e.g. [2], [9]).

Since classical anatomical methods require the sacrifice of the subjects, subsequent electrophysiological investigation of the functional properties of anatomically identified areas in the same subjects is impossible. Such an approach would be advantageous, considering the demonstrated inter-individual variability in the location of brain areas. One commonly used method has been the mapping of connected neurons in living animals using electrophysiological techniques that apply electrical stimulation via a microelectrode inserted into one brain area and the identification of antidromic or orthodromic responses recorded by a second microelectrode in another [10]. This technique has provided important insights into the discharge properties of neurons identified as input or output neurons [11]–[15]. Unfortunately, it can only identify connected neurons at the tips of the microelectrodes and is therefore not useful for mapping neural connectivity on a large scale. This same problem applies to optical imaging, which has also been used to obtain high-resolution functional maps of relatively small areas on the brain surface for subsequent electrophysiological characterization [16].

Another promising technique for mapping large scale neuroanatomical connections *in vivo* is the injection of MRI-visible contrast agents such as manganese (Mn^2+^) into a particular brain region [17]. Manganese is transported mainly anterogradely and also transsynaptically. This technique has been used to map neural connections in primates [18], but requires careful attention to dose, as Mn^2+^ is cytotoxic [17].

Several researchers have used cortical cooling of one brain area [19], [20] and then measured the impact on other areas using the 2-deoxyglucose (2DG) technique. This technique offers a way of measuring the functional impacts of connections but it also requires the sacrifice of the animals.

BOLD based fMRI has become the tool of choice for large-scale non-invasive mapping of brain function. Neural networks can be mapped non-invasively using localized changes in the regional cerebral blood volume (CBV), flow (CBF) and oxygenation which create modulations of the signal intensities in BOLD images [21], [22]. These changes can subsequently be used to produce statistical maps that identify task-related brain regions. In addition, several techniques have been developed to generate functional connectivity maps independent of experimental tasks [23]–[25]. This approach is very promising but also indirect as the outcome is dependent on the models that are employed [26].

Recently, several studies have used concurrent repetitive transcranial magnetic stimulation (rTMS) and positron emission tomography [27]–[29] or fMRI [30] to study cortico-cortical connectivity in human subjects. These studies found that rTMS modulated the activation at both the stimulation site and in several distant cortical areas that were presumably connected with the stimulated cortex. Thus, the combination of stimulation with modern imaging techniques may be a particularly potent methodology for the non-invasive mapping of the functional connectivity of the brain. Indeed, Tolias and coworkers demonstrated recently that electrical microstimulation elicts reliable BOLD activations at the tip of a microelectrode in the visual cortex of anaesthetized monkeys [31]. Moeller and colleagues recently used concurrent fMRI and microstimulation to reveal a face-selective network of cortical and subcortical areas in monkeys [32]. Here, we further explored the viability of using concurrent electrical microstimulation and fMRI to study neural connectivity in living monkeys. To evaluate this technique, we studied the effects of microstimulation in the midbrain superior colliculus (SC), a critical node of the saccadic eye movement system, the best understood sensory-motor system in primates [33], [34]. The intermediate layers of the SC form a motor map for the rapid movements of the eyes that shift gaze to objects of interest. Neurons in the intermediate layers receive afferents from many cortical and subcortical areas and project directly to preoculomotor neurons in the brainstem. The SC also projects back to the cortex via the thalamus. Our results demonstrate that a short microstimulation pulse train delivered to the SC results in BOLD signal changes in the SC and in cortical and subcortical areas that are known to be connected with the SC, suggesting that combined microstimulation and fMRI may indeed be a useful methodology for the investigation of neural connectivity in living animals.

## Methods

### Preparation of the animals and identication of the stimulation sites

Two male rhesus monkeys (M1 and M2) (*Macaca mulatta*) were subjects in the present experiment. All experimental methods described were performed in accordance with the guidelines of the Canadian Council on Animal Care policy on the care and use of experimental animals, and an ethics protocol approved by the Animal Users subcommittee of the University of Western Ontario Council on Animal Care.

Animals were prepared for the experiments by undergoing a surgical procedure to place an MRI-compatible implant on their skull. A plastic chamber (Crist Instruments, Hagerstown, MD) was centered on the midline and tilted 38 deg posterior of vertical to allow recordings and microstimulation in the SC. The chamber and a plastic head post (Crist Instruments, Hagerstown, MD) to restrain the head were anchored to the skull with 6 mm ceramic bone screws (Thomas Recording, Giessen, Germany) and dental acrylic. The SC was initially localized with anatomical MR images. The monkeys were trained to perform a fixation task and the intermediate layers of the SC were identified through single neuron recordings (Plexon MAP system, Plexon, Texas) and constant current microstimulation (Grass S88 with PSIU6 photoelectric stimulus isolation unit, Astro-Med, RI) using previously described criteria [35]–[37]. Eye movements were recorded at 120 Hz using an infrared eye tracking system (ISCAN, Burlington, MA).

### Placement of the microelectrodes

On the day prior to each experiment, one or two commercially available monopolar epoxy-coated tungsten microelectrodes (UEWLFELMNN1E, 200 µm shank diameter, 140 mm length, Frederic Haer, Bowdoinham, ME) were lowered into the intermediate layers of the left SC by a hydraulic microdrive (Narashige, Tokya, Japan) through 23 gauge stainless steel guide tubes that were held in position by a plastic grid inside the recording chamber. The impedances of the microelectrodes ranged between 150 to 500 kΩ at 1000 Hz. The animals were slightly sedated with ketamine hydrochloride (1 mg/kg im) to minimize movement during the procedure. The correct location of the microelectrodes was verified by established criteria: electrodes 1–3 mm below the dorsal surface of the SC, characteristic discharges for contralateral saccades, microstimulation evoked saccades at a low threshold (<50 µA at 100 ms, 300 Hz, 0.3 ms biphasic pulse trains). The guide tubes were then slowly pulled out using a pair of hemostats while the electrodes were still attached to the microdrive. The microelectrodes were then bonded to the plastic grid inside the cylinder with epoxy glue (Copper Bond Epoxy Glue, the Noble Company, Grand Haven, MI). The microelectrodes were then cut ∼10 mm above the grid with a small wire cutter. The wire cutter was sufficiently sharp to ensure that the cutting procedure did not disturb the electrode placement. The insulation was removed with a scalpel at the end of the electrode (∼5 mm). A gold Amphenol connector was then crimped on to the newly cut end. Following this, several microstimulation pulse trains were delivered and eye movements were examined to verify that the electrodes had not moved during this procedure.

### Preparation of the imaging and stimulation session

Prior to the experimental session, the animal was anesthetized with propofol (0.2 mg/kg/min IV) and placed in an MR compatible horizontal primate chair and positioned within a whole body 4 Tesla MRI system (Varian, Palo Alto, CA; Siemens, Erlangen, Germany). A 12 cm transmit-receive cylindrical saddle primate head coil was used for transmission and detection of signal and the animal's head was immobilized within the RF coil using an acrylic head post. Immediately prior to the imaging experiments, microstimulation pulses (200 ms pulse trains, 300 Hz, 0.3 ms biphasic pulses) were applied using a microstimulator with a photoelectric stimulus isolation unit (Grass S88 with PSIU6 photoelectric stimulus isolation unit, Astro-Med, RI). This constant-current microstimulator and isolation unit were placed in the control room and connected with the microelectrodes by a 30 m coaxial BNC cable. The animal's eyes were propped open with a plastic eyelid retractor and the eye positions of the anesthetized animal were visually monitored. The current threshold for the fMRI experiment was selected so that saccadic eye movements were evoked on every stimulation trial in the anesthetized animal. All sites in the caudal SC evoked saccades >8° which were easily detected by observing the monkeys' eyes. For those experiments in which we used an additional electrode in the rostral SC, we used the same level of current that evoked saccades at the caudal site. The necessary stimulation currents for evoking saccades at 300 Hz ranged from 250–400 µA in the anesthetized monkey compared to currents <50 µA (via a 40 cm cable) in the awake animal the day before. Microstimulation at 150 Hz did not evoke saccades at this current level. To ensure that this elevation in stimulation threshold in the anesthetized compared with the awake monkey was not due to the capacity of the cable used during experimental sessions, we examined the current required to elicit a saccadic eye movement after SC microstimulation using a 30 m cable and a 40 cm cable in an awake animal. We found that only a 30% increase in current was necessary to evoke saccades with the 30 m BNC cable compared with the 40 cm cable. Thus, the elevation in stimulation threshold we observed during the imaging experiments is most likely due to the known GABA_A_ mediated suppression of neural activity by propofol [38]–[40]. After two of the imaging sessions, we stimulated three days later again through the implanted electrode in the awake monkey and confirmed in both cases that the stimulation threshold had returned to currents of less than 50 µA.

To prevent any visual input after an evoked saccade, the animal's eyes were closed and occluded by wrapping vetrap (3M, London, Ontario, Canada) three times around the monkey's head in front of his eyes just prior to the start of the imaging experiments. This procedure had the added benefit of minimizing any potential ear movements in the animals. The lights were also turned off in the magnet room.

### Microstimulation concurrent with fMRI

**Figure 1:**
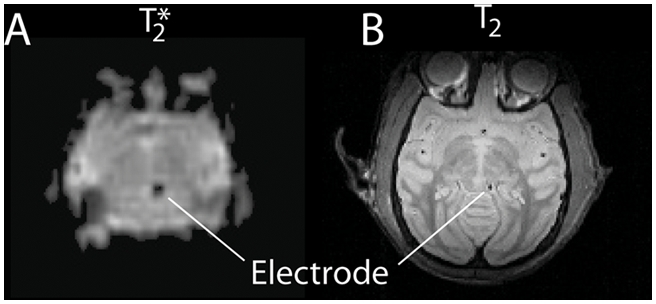
Artifact created by the microelectrode. T2* (a) - and T2-weighted images (b) show the extent of the artifact created by the microelectrode in the functional and anatomical images.

Imaging planes for the functional scans were prescribed from a series of sagittal anatomic images acquired using T1-weighting. Fifteen contiguous functional planes, 2.5 mm thick, were prescribed axially to the monkey brain extending from the superior extent of the cortex covering the entire brain. This slice plane was chosen to cover the cortex and brainstem with the smallest number of slices possible. A constrained, three-dimensional phase automatic shimming procedure was employed to optimize the magnetic field homogeneity for improving the signal-to-noise ratio and to reduce artifacts over the prescribed functional planes [41]. During each functional task, BOLD images (T_2_*-weighted) were acquired continuously using an interleaved, two segment, optimized EPI imaging protocol (64×64 matrix size, TR = 1000 ms, TE = 15 ms, flip angle = 40°, 12.8 cm FOV, volume collection time = 2 s, EPI voxel size of 2×2×2.5 mm). Each image was corrected for physiological fluctuations (cardiac, respiration) using a global navigator echo correction [42]. The tungsten microelectrodes created only small artifacts in their immediate vicinity (see Fig. 1). No artifacts or distortions were observed by the copper wires leading to the electrodes. For each event-related functional run, a 200 ms current pulse train (150 or 300 Hz, 0.3 ms biphasic pulses, 250–400 µA depending on the previously determined saccade threshold) was passed through the implanted electrode and an indifferent electrode placed in the chamber every 12 s. Each 3 min run started with a 12 s no stimulation period and contained 14 pulse trains with an inter-stimulus interval of 12 s, yielding a total of 90 volumes (6 volumes (a single volume being 2 s) at the beginning plus 14 times 6 volumes (12 s)) per run. A TTL pulse from the imaging control computer synchronized microstimulation pulses to the imaging volume times.

During each of the 5 experimental sessions (2 for m1, 3 for m2), 6–20 runs were obtained along with a T_2_-weighted anatomic reference volume acquired along the same orientation and field of view as the functional images using a fast spin echo (FSE) acquisition scheme (256×256 matrix size, 35×1.25 mm slices (0.5 mm×0.5 mm in-plane resolution), TR = 6 s, ESP = 16.0 ms, ETL = 8, Eff_echo = 1).

### Data Analysis

Analyses were conducted using BrainVoyager QX version 1.3 (Brain Innovation, Maastricht, The Netherlands). BrainVoyager resamples functional runs into 1.5×1.5×1.5-mm voxels. Functional data were superimposed on the anatomical scans for each monkey. All functional image series underwent 3D motion correction and temporal filtering (linear trend removal, high-pass filter with a cut-off of 0.0389 Hz (7 cycles per run), Gaussian filter in time domain with full width at half-maximum (FWHM) of 4 s). Functional data were statistically analyzed using the general linear model (GLM) framework. To construct the predictor curves for the microstimulation pulses, we defined a boxcar function representing the alternating stimulation protocol (microstimulation = 1 (one volume), no stimulation = 0 (5 volumes)), and then convolved the function initially with Brain Voyager's model of the hemodynamic transfer function using parameters delta = 2.5 s and tau = 1.25s [43].

We found that the mean BOLD signal time course in many areas, including in the superior colliculus, was different from Brain Voyager's hemodynamic transfer function. We therefore used the mean z-scaled evoked BOLD response in the superior colliculus as the reference time course function in our analysis (see Figure S1). We obtained this function by averaging the BOLD response at the tip of the microelectrode across multiple sessions from both monkeys. This transfer function resulted in more extended activations in the superior colliculus and in cortical areas.

Data were corrected for serial correlations and z-scaled (mean removed and divided by SD) on a run-by-run basis to remove arbitrary signal amplitude effects. This GLM analysis resulted in statistical contrast activation maps which were corrected for multiple comparisons using the statistical false discovery rate implemented in Brainvoyager QX [44] and thresholded at the corrected P<0.05. We included in our analysis only those activated regions with volumes ≥25 mm^3^ (2.5 original voxels).

Activation peaks were identified in individual subject maps and are reported relative to the anterior commissure. Mean BOLD signal time courses for each region identified were computed to illustrate how the BOLD signal evolved in the response to the 200 ms microstimulation pulses. Raw data from each trial were transformed into percentage signal change values, [(signal – baseline)/baseline]* 100, where baseline was defined as the activation in the 1s volume before microstimulation on a trial-by-trial basis. Activated regions were identified and labeled using a standard atlas of the rhesus monkey brain [45]. Anatomical images for one monkey were segmented at the gray/white matter boundary and rendered for visualization purposes [46].

## Results

### Caudal SC Microstimulation at 300 Hz

**Figure 2:**
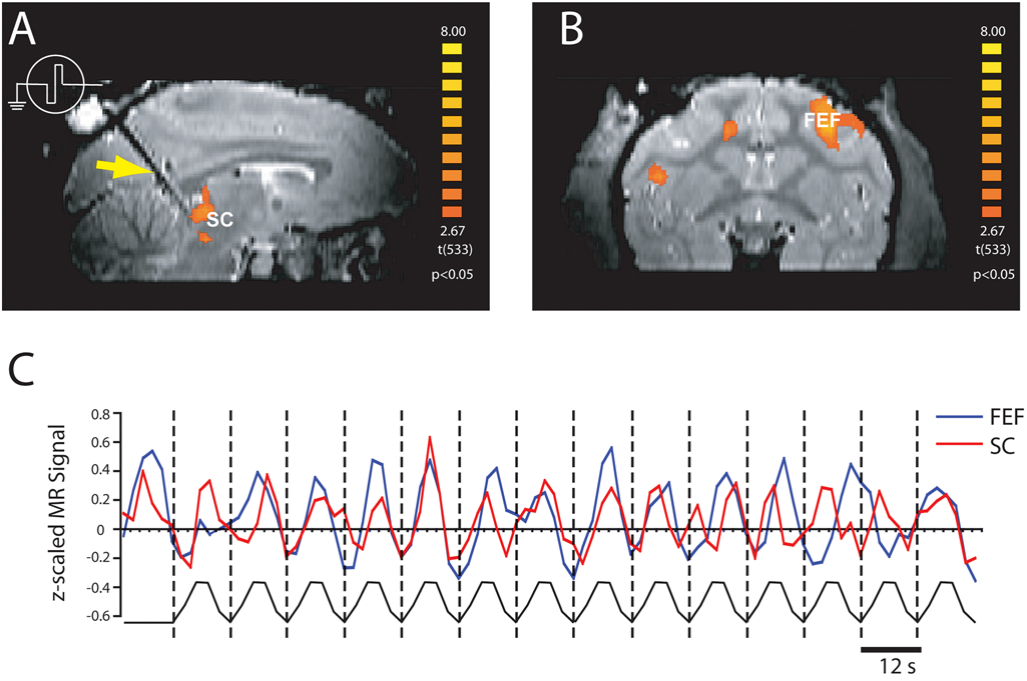
Activation maps and activation time courses for SC and FEF activated regions in subject m1 (session 1). (a) SC activation map is superimposed on a parasaggital section of structural T2-weighted MRI (p<0.05, corrected for multiple comparisons). An increased BOLD signal in the SC at the tip of the electrode (dark artifact in the MRI, yellow arrow) evoked via microstimulation is clearly visible. (b) Coronal section at the level of the FEF with superimposed activation map ((p<0.05, corrected for multiple comparisons) from the same session. (c) Activation time courses for the SC (all activated voxels in a 28.6 mm^3^ volume at the tip of the microelectrode) and FEF (all activated voxels in a 112mm^3^ volume) from one 3 min run. Dashed lines indicate the time of 200 ms microstimulation.

**Figure 3:**
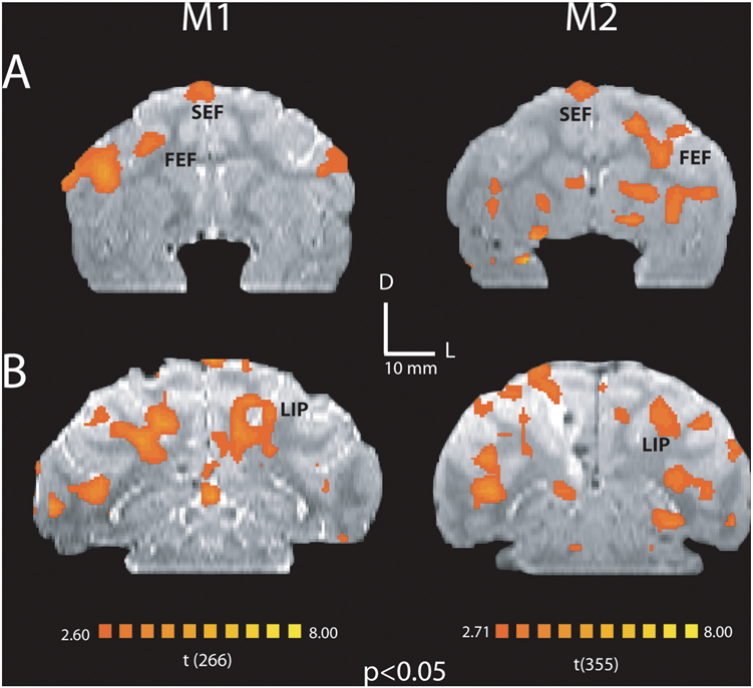
Activation maps show similar BOLD activity in (a) FEF, SEF, and (b) LIP across subjects, m1 (session 2) and m2 (session 4), following caudal SC microstimulation. Activation maps are superimposed on coronal sections of structural T2-weighted MRI (p<0.05, corrected for multiple comparisons). The anterior-posterior coordinates relative to the anterior commissure for each section are (a) 1 mm and 4 mm, and (b) -21 mm and -20 mm for subjects' m1 and m2, respectively.

Microstimulation evoked a clear activation in the SC at the tip of the microelectrode (Fig. 2A), confirming that the current pulse train was able to elicit changes in BOLD based MRI signal intensities. In all sessions, the SC was consistently the strongest activated brain region and its BOLD activity correlated with the stimulus time course (Fig. 2C). Modulations were also observed in the MRI signal in many cortical and subcortical areas that are known from previous anatomical studies to be connected with the SC, supporting the idea that microstimulation of the SC leads to neural activity changes in connected areas. The frontal eye field (FEF), a frontal brain region implicated in top-down control of saccadic eye movements [47], showed BOLD activation from caudal SC stimulation (Fig. 2B) and an activation time course that also correlated well with the stimulation pulses (Fig. 2C). In addition, we observed modulations of all other cortical areas previously defined as saccade-related following caudal SC stimulation in both monkeys. Figure 3 shows activation in a variety of cortical areas in both monkeys, including FEF, supplementary eye fields (SEF) and lateral intraparietal area (LIP) in M1 and M2. A total list of activated areas for monkey M1 and M2 are given in Table S1 and S2, respectively.

**Figure 4:**
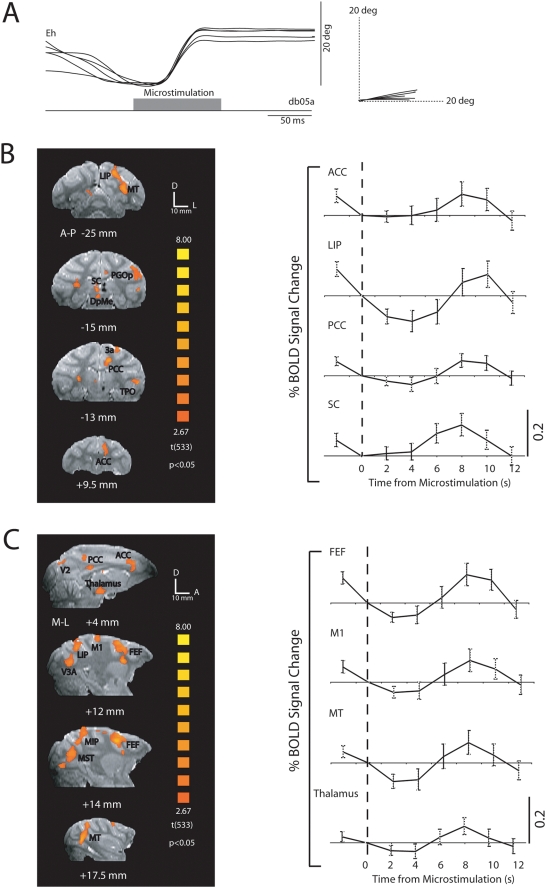
Effect of caudal SC microstimulation on cortical and subcortical BOLD signals in subject m1 (session 1). (a) Eye-position traces (filtered with a 30 Hz low-pass filter implemented in Matlab) evoked by microstimulation (40 µA) of caudal SC. 100 ms stimulation of the left SC produced 20° rightward saccades. Traces were obtained immediately before the microelectrode was glued in place. Activation maps (84 trials, six 3 min runs with 14 stimulations each) are superimposed on coronal (b) and parasaggital (c) sections of structural T2-weighted MRI (p<0.05, corrected for multiple comparisons). The numbers below each image indicate the anterior-posterior (A-P) coordinates (b) and the medial-lateral (M-L) coordinates (c) relative to the anterior commissure. Average BOLD signal time courses in response to the 200 ms current pulse train (84 trials, six 3 min runs with 14 stimulations each) for activated regions of interest localized in the coronal and parasaggital slices. Percentage BOLD signal change plotted by time in seconds (s). Error bars represent the standard error of the mean across trials. ACC, anterior cingulate cortex; DpMe, deep mesencephalic nucleus; Eh, horizontal eye position, FEF, frontal eye fields; LIP, lateral intraparietal region; M1, primary motor cortex; MST, medial superior temporal area; MT, middle temporal area; PCC, posterior cingulate cortex; PGOp, parietal area PG, opercular part; SC, superior colliculus; TPO, temporal parietooccipital associated area in sts; V2, visual area 2; V3A, visual area 3A.

**Figure 5:**
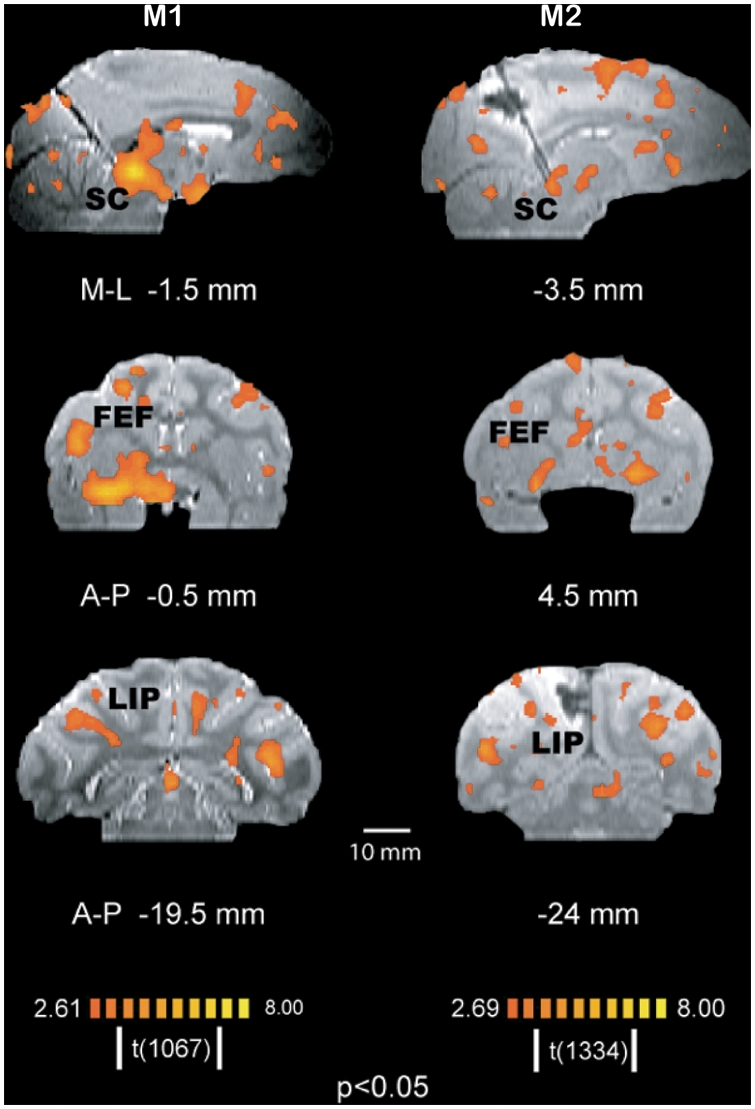
Activation map across sessions in subject m1 (average of 12 3-minute stimulation runs) and subject m2 (average of 15 3-minute stimulation runs). Activation maps are superimposed on parasaggital and coronal sections of structural T2-weighted MRI (p<0.05, corrected for multiple comparisons). The numbers below the images indicate the medial-lateral (M-L) coordinates (parasaggital slices) and anterior-posterior (A-P) coordinates (coronal slices) relative to the anterior commissure for each subject. FEF, frontal eye fields; LIP, lateral intraparietal region; SC, superior colliculus.

Figure 4 shows the extent of cortical and subcortical BOLD activation that was evoked by stimulation of a site in the left SC of monkey m1 that elicited ∼20° horizontal rightward saccades (Fig. 4A). We found BOLD signal changes in several visual areas (V1, V2, V3, MT/MST) (Fig. 4C), in the somatosensory cortex (area 1, 2, 3a/b) (Fig. 4B) and in the primary auditory cortex. Microstimulation also evoked BOLD-signal activations in the anterior (ACC) and posterior (PCC) cinguli, the lateral intraparietal (LIP) area and the primary motor cortex (M1) (Fig. 4B,C). The fact that BOLD activity was also found in the thalamus (Fig. 4B,C) suggests that the observed cortical BOLD signal changes may be in part due to orthodromic thalamocortical projections and not antidromic connections alone. The SC BOLD signal time course shows a gradual rise for the first few seconds, after which there is a sharp increase that peaks at 8s from stimulation and declines back down to baseline by 12s (Fig. 4b). Cortical areas show a slightly different trend in which their BOLD signal initially decreases below baseline before it steadily increases and peaks between 8–10s from stimulation. It is likely that this initial drop of the BOLD signal below baseline is a carry-over effect from the previous stimulation 12 s earlier that was still returning to baseline at the time of microstimulation and not a negative BOLD response. If we look at activity averaged across all experimental sessions for both M1 and M2 (Fig. 5) a strong BOLD signal can be seen in SC, as well as strongly connected areas including ipsilateral FEF and LIP. Both monkeys showed similar activated areas.

### Experimental Sessions

**Figure 6:**
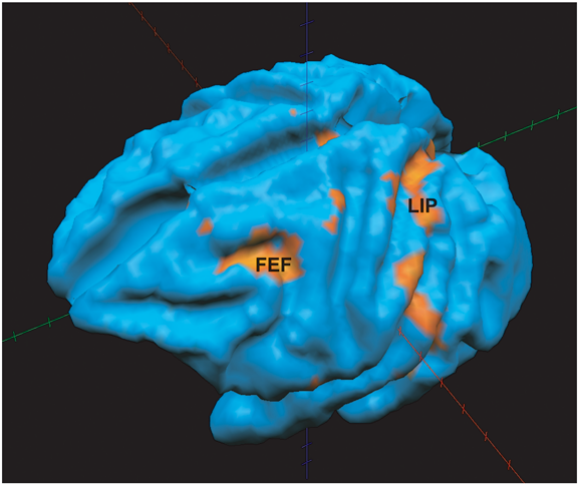
FEF and LIP 3D BOLD activation after caudal SC microstimulation in subject m1 (session 1). Functional activation map (p<0.05) is rendered on the subject's brain segmented at the grey/white matter boundary.

Table 1 provides a parametric outline of each of the stimulation sessions that were conducted. Eight experiments were run in total: 3 conducted in M1 (2 caudal stimulations, 1 rostral stimulation) and 5 in M2 (3 caudal stimulations, 2 rostral stimulations). In each experimental session, the stimulation site in the SC was determined by the amplitude and direction of the evoked saccades. These were calculated from eye-position traces recorded prior to gluing the electrode into the SC. In both monkeys, the left SC was stimulated and therefore rightward saccades were generated. Caudal stimulation sites produced mainly horizontal saccades with amplitudes ranging from 8–22°, though these eye movements sometimes contained a slight vertical component. Rostral stimulation generated horizontal saccades with an amplitude of approximately 1°. In one case where fixation-related neural activity was recorded, stimulation produced no detectable saccades but was found to suppress voluntary saccades. BOLD-activation of the left SC was observed in all 8 sessions. BOLD signal modulations also occurred in both the ipsilateral frontal eye field (FEF) and lateral intraparietal (LIP) area for the majority of the experiments. FEF and LIP are two of the major cortical areas shown to project to the intermediate layers of the SC and thought to be involved in saccade generation [33]. A three-dimensional rendered brain with a superimposed BOLD activation map for subject M1 is shown in Fig. 6. This 3D image shows that microstimulation-evoked activation of the arcuate region was localized in the fundus and anterior wall of the sulcus, consistent with the known location of the frontal eye fields in monkeys [48]. In addition, we found activation in the lateral intraparietal sulcus consistent with the known location of area LIP [49].

**Table 1:** SC microstimulation experimental sessions.

Session	Subject	Evoked Saccade	Saccade Threshold (µA)^a^	SC			FEF ipsilateral			FEF contralateral			LIP ipsilateral			LIP contralateral		
				x	y	z	x	y	z	x	y	z	x	y	z	x	y	z
1	M1	13°R, 2°U	40 (400)	−2	−15	−1.5	−13	3	15.5	14	0.5	14	−10.5	−24	15	10.5	−25	16
2	M1	b		−4	−17.5	−4.5	−21.5	−1	10.5									
	M1	8°R, 0.5°D	30 (400)	−1.5	−18.5	−2	−12	0.5	11.5	14	−0.5	11.5	−9	−24	13	11.5	−21	13.5
3	M2	8.5°R	20 (300)	−3	−14	−5.5	−16	1	15	16	0.5	15.5	−8.5	−20.5	9	7	−28.5	15
4	M2	1°R, 0.5°U	30 (350)	−3.5	−15	−4.5							−7.5	−24	11	7	−26	12.5
	M2	22°R, 3°U	15 (250)	−2	−13.5	−1.5	−16	2.5	13	12.5	5	10	−13	−21	14.5	13	−20	14.5
5	M2	1°R	30 (300)	−1.5	−17.5	−3.5	−13.5	3.5	10.5	22	4.5	8						
	M2	28°R, 5°U	30 (300)	−2.5	−17	−7	−13.5	5.5	11									

Significant regions at a voxel level of p<0.05 corrected for multiple comparisons. Coordinates (mm) are given in relation to the anterior commissure. D, downward; FEF, frontal eye field; LIP, lateral intraparietal area; R, right; SC, superior colliculus; U, upward.

a Numbers indicate the saccade threshold in the awake animal and numbers in brackets indicate the saccade threshold in the anesthetized animal.

b No detectable saccade was evoked at this site but we recorded fixation-related activity. The stimulation current during the imaging session was set to 400 µA (same as at the caudal site in the same session).

Although we observed BOLD signal changes in the hemisphere ipsilateral to the SC stimulation sites, we also found activation in the contralateral hemisphere in all sessions. Two areas in the contralateral hemisphere that were modulated in most sessions were FEF and LIP. However, there were no sessions in which BOLD activity in the contralateral FEF or LIP changed without a concomitant signal change in the ipsilateral hemisphere, a finding consistent with the known ipsilateral projections of these cortical areas to the SC [2], [50].

**Figure 7:**
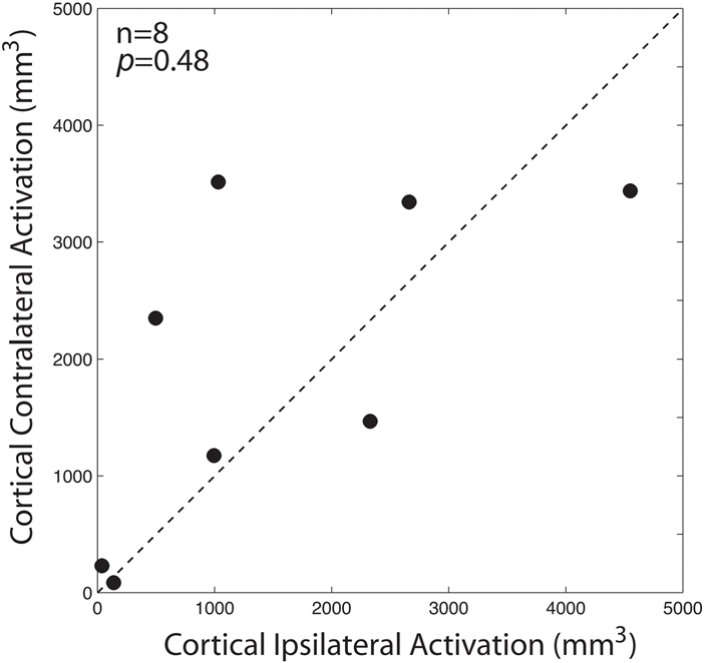
Effect of SC microstimulation on cortical activation. The total volume of cortical activation in the hemisphere ipsilateral to the SC stimulation site (abscissa) is plotted against the cortical activation in the hemisphere contralateral to the stimulation site (ordinate). Each dot represents the cortical activation for one microstimulation site. The dashed line represents the unity line (slope = 1).

To quantify this laterality effect, we measured the total activated volumes in each hemisphere for each of the 8 experiments. In Fig. 7 we have plotted the total ipsilateral activation against the total contralateral activation for each experiment (filled circles). This analysis did not show any cortical laterality effect of SC stimulation. Stimulation evoked significant increases in the BOLD signal in 1520 (±551 SEM) mm^3^ of the ipsilateral hemisphere and 1842 (±497 SEM) mm^3^ of the contralateral hemisphere (p = 0.48, paired t-test).

### Effect of different stimulus frequencies

**Figure 8:**
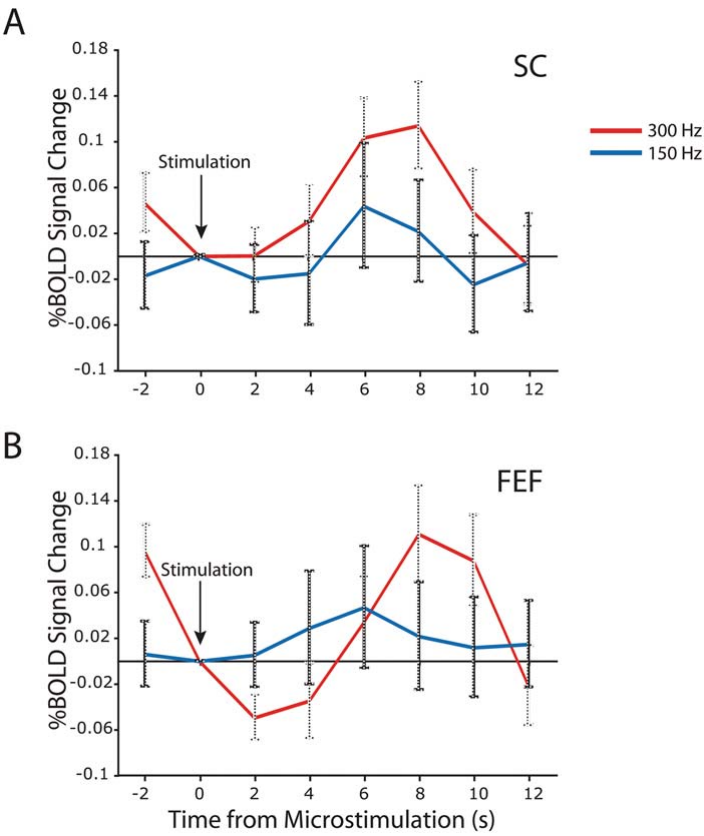
Effect of stimulation frequency on BOLD signal changes in subject m1 (session 1). (a) Average BOLD signal time course (84 trials, six 3 min runs with 14 stimulations each) in response to 300Hz current pulse trains (red) and 150Hz current pulse trains (blue) in the stimulated caudal SC. (b) Same as (a) for FEF. Percentage BOLD signal change plotted by time in seconds (s). Error bars represent the standard error of the mean across trials.

To investigate the relationship between microstimulation frequency and evoked BOLD responses, we compared individual subject maps for the 150 Hz and the 300 Hz microstimulation conditions. Microstimulations at 300 Hz evoked saccades whereas the 150 Hz stimulation trains did not elicit saccadic eye movements. The microstimulation trains at 300 Hz evoked activation in many cortical and subcortical areas, whereas 150 Hz trains evoked very few significant activations in either monkey for the significance criteria that we employed. We next created event-related averages for selected regions of interest that were identified in the 300 Hz pulse train condition. Fig. 8 shows event-related time courses for the SC and FEF for both stimulation conditions. The 300 Hz pulse trains evoked a significantly stronger BOLD response in both the SC and FEF than the 150 Hz pulse trains. At 300 Hz, the BOLD signal peaked at 8 s and then decayed back to baseline at 12 s in both areas following microstimulation. Conversely, the 150 Hz BOLD response in both SC and FEF peaked at 6s following microstimulation.

## Discussion

We have shown here that neural networks can be identified by combining microstimulation and fMRI. BOLD signal changes in a number of cortical and subcortical areas consistent with the previously identified connectivity of primate SC were modulated via short 200 ms microstimulation pulse trains. The FEF, located in the anterior bank of the arcuate sulcus [48], the SEF, found on the medial wall of the dorsofrontal cortex [51], and area LIP all project directly to the caudal SC and have a role in the generation of saccades. The ACC also projects directly to the SC [2] and, in fMRI studies, has shown an increase in BOLD-related activity for voluntary saccades [52]. Histological studies have previously established collicular projections from the various sensory cortical areas, including the primary auditory cortex and other auditory areas, the primary and premotor regions, the somatosensory cortices, and the primary visual cortex and higher order visual cortices, such as V2, V3, and MT/MST [50].

A number of subcortical regions were also activated via SC stimulation. Of particular interest are the activations in the pulvinar and in the mediodorsal nucleus of the thalamus, areas known to intermediate between the SC and cortex [13], [53]. This result raises the possibility that the cortical activations were at least in part evoked by orthodromic activations from the SC to the cortex via the thalamus. At the present, it is not possible to distinguish between ortho- and antidromically activated areas with this technique.

A surprising finding was the absence of any clear laterality effect of SC microstimulation on cortical activation. Retrograde labelling studies have found very little contralateral projections to the SC [2], [50], [54]. Several mechanisms could account for the extended activation in the contralateral hemisphere in this study. First, microstimulation might have evoked activation in the contralateral SC due to intercollicular connections [55]. Indeed, we found in some sessions BOLD activations in the contralateral SC, although it was much lower than at the tip of the microelectrode. Second, activated cortical areas might have activated the contralateral hemisphere through the corpus callosum. It has been directly shown that microstimulation of FEF leads to activity changes (both excitatory and inhibitory) in the contralateral FEF in about 50% of the neurons [56]. Austin et al. [57] showed that direct microstimulation (50 ms pulse trains: 0.3 ms pulses, 300 Hz, ∼2.3mA) of the rat motor cortex evoked a significant increase in BOLD-activity in both stimulated and contralateral cortices and in some experiments a higher percent signal change in the contralateral motor cortex. Ekstrom and colleagues found that electrical microstimulaiton of the FEF evoked an increased fMRI signal in the FEF in the other hemisphere. It should also be noted here that a crossed projection from the SC to the FEF (through the thalamus) has been demonstrated recently. Crapse and Sommer reported that a large proportion of orthodromically activated FEF neurons (22/53) were activated following microstimulation in the contralateral SC [58]. We cannot rule out that the absence of clear laterality effects were due to unnatural high levels of stimulation currents (250–400 µA) in this study. Although these high currents were necessary to evoked saccades in the anaesthetized animals, it would be worth investigating whether lower stimulation currents could be used to obtain more lateralized activation patterns in future studies.

It is clear that the SC has connections to many more areas than we identified in these stimulation experiments. A problem which plagues all fMRI studies is the fact that the number of activated voxels depends on the significance criteria that are applied. We chose relatively conservative criteria by correcting for serial correlations, performing corrections for multiple comparisons, and by applying a minimal cluster size of 25 mm^3^. The activated regions therefore represent only the regions in which the evoked BOLD signal best fit the predictor curve. These regions are almost certainly not the only areas which were modulated by the microstimulation pulses. While it is tempting to speculate that these regions represent the areas with the strongest connections to the SC, factors beyond the strength of neural connections might influence the BOLD signal. One likely factor is anesthesia, which is known to have different effects on the BOLD signal in different brain regions [59]. In addition, differences in the strength of the BOLD response might also be related to differences in the vascular beds between brain regions [60].

Anatomical images explicitly demonstrated that the electrode tip was placed in the SC of both subjects. BOLD activation in the SC was greatly increased following microstimulation with 300 Hz pulse trains suggesting that the SC neurons were activated around the tip of the electrode leading to a signal change in connected areas. Based on our results, we assume that both antidromic and orthodromic projections were activated here. The fact that 150 Hz pulses evoked drastically reduced activation compared with the 300 Hz stimulation indicates a relationship between evoked neural activity and the BOLD response.

We believe that the increased BOLD responses at the tip of the microelectrode in the SC were predominately if not entirely the result of neuronal-BOLD coupling and not of vasomotor responses evoked by direct stimulation of arteriole smooth muscles. Direct electrical stimulation of blood vessels leads to vasoconstriction [61] and the long chronaxie of smooth muscles [62] makes it impossible to stimulate smooth muscles without stimulating neurons first.

The neural processes underlying the BOLD signal obtained in fMRI experiments are still poorly understood. Recent experiments have concluded that the BOLD response is slightly better correlated with local field potentials than single- or multi-unit activity, suggesting that synaptic processes contribute more to the BOLD signal than neuronal firing [22]. We believe that the concurrent use of fMRI and microstimulation may provide an important tool to further our understanding of the physiological basis of the BOLD-signal by allowing for a controlled modulation of neural activity. We only compared two different stimulation frequencies here and observed stronger BOLD responses in the SC for 300 Hz than 150 Hz. Further studies that systematically change the parameters within the microstimulation train and analyze the effect on neural activity and the BOLD-response in a connected area could reveal important information about the relationship between neural activity and BOLD fMRI.

Most importantly, this technique can be used to identify key areas for future electrophysiological studies in the same animal. This technique therefore might be used in addition to fMRI experiments in awake, behaving monkeys. Indeed, two recent studies have used fMRI in awake, behaving monkeys trained to perform visually-guided saccades to map saccade-related areas in nonhuman primates [63], [64]. The activation maps that were obtained in these studies are very similar to those that we obtained in response to SC microstimulation, supporting the notion that combined microstimulation and fMRI is a potentially useful method for the study of functional connectivity.

Taken together, the results reported here describe a technically relatively simple procedure using microstimulation combined with fMRI to map the connectivity of the SC in living monkeys. Tolias et al. have shown recently that microstimulation in V1 leads to an increased BOLD signal in MT/V5 [31] and Ekstrom and colleagues have demonstrated that microstimulation of FEF leads to an increased fMRI signal in several higher-order visual areas and in the SC [65]. Moreover, Moeller et al. have demonstrated that microstimulation in the face-selective patches in the ventral stream activates a characteristic network of cortical and subcortical areas [32]. We therefore believe that this technique can also be applied to map neural connectivity in other motor or sensory systems, thereby providing an important new tool for the identification of connected brain areas. In addition to shedding light on neural connectivity in animal models, the same approach could be of use in fMRI studies of human patients to investigate the mechanisms involved in deep brain stimulation for tremor reduction in Parkinson's disease [66] or in electrical stimulation for the alleviation of depression.

## Supporting Information

Figure S1 Comparison of Boynton's hemodynamic response function (HRF) and empirical HRF.
(0.24 MB EPS)

Table S1 (0.18 MB DOC)

Table S2 (0.13 MB DOC)
