# Supplementary material for: Connectivity of the Primate Superior Colliculus Mapped by Concurrent Microstimulation and Event-Related fMRI
Source: PLoS One. 2008 Dec 11;3(12):e3928. doi: 10.1371/journal.pone.0003928 (PMC2592545; doi:10.1371/journal.pone.0003928)
Supplement: Table S1 — (0.18 MB DOC) [file pone.0003928.s002.doc]

Table S1. BOLD-modulated brain areas in monkey m1

|  |  |  |  |  |
| --- | --- | --- | --- | --- |
|  | Hemisphere |  | Coordinates |  |
|  |  | D-V | M-L | A-P |
| Frontal Cortex |  |  |  |  |
| ACC | L | 15 | -3.5 | -4 |
|  | R | 9.5 | 1 | -2.5 |
|  | R | 15 | 5 | -0.5 |
| ACC area 23b/c | R | 12.5 | 1.5 | -7 |
| ACC area 23c | R | 12.5 | 6.5 | -8.5 |
| ACC area 24a/b | R | 9 | 4.5 | 17 |
| Area 14, medial part | Mid | 3 | 0 | 18.5 |
| Area 46 | R | 8.5 | 11 | 17 |
| FEF | R | 15.5 | 16 | 0.5 |
|  | L | 15 | -16 | 1 |
|  | L | 12.5 | -14 | 4.5 |
|  | R | 13 | 16 | 4.5 |
|  | R | 7 | 21 | 8 |
| M1 | L | 21.5 | -11 | -9.5 |
|  | L | 15 | -15.5 | -7 |
|  | R | 19.5 | 12.5 | -7 |
|  | L | 20.5 | -11.5 | -6 |
| PFC | R | 10 | 18 | 12 |
| PM | L | 10 | -20.5 | -4.5 |
|  | R | 15 | 20.5 | -2 |
| SEF | Mid | 20 | 0 | -0.5 |
| Parietal Cortex |  |  |  |  |
| ACC area 31 | L | 11.5 | -2 | -16 |
| LIP | L | 9 | -8.5 | -20.5 |
|  | L | 13.5 | -23.5 | -11.5 |
| MIP | R | 17.5 | 8 | -22 |
| MIP/PEC | R | 19.5 | 15.5 | -21.5 |
| PE | R | 19.5 | 10.5 | -19 |
| PEC | L | 20 | -10.5 | -20 |
|  | R | 19.5 | 10.5 | -19 |
| PECg | R | 18.5 | 2.5 | -18 |
| PGM | L | 11.5 | -1 | -23.5 |
|  | R | 17.5 | 3.5 | -22 |
| S1 | R | 21.5 | 10.5 | -11.5 |
| S2 | R | 21.5 | 10.5 | -11.5 |
|  | L | 5.5 | -23 | -10.5 |
| S2E | L | 4.5 | -27 | -6 |
| Temporal Cortex |  |  |  |  |
| AKL | R | 7 | 28 | -12 |
|  | L | 6 | -26.5 | -10 |
|  | R | 5 | 27.5 | -10 |
|  | L | 1 | -27 | -6 |
|  | R | -2 | 28.5 | -4 |
| Insula | L | -4.5 | -19 | 1.5 |
|  | L | -3.5 | -22 | 3.5 |
| PaI | L | -7 | -19 | -3.5 |
| PGa | L | -2.5 | -20.5 | -13 |
|  | L | -9 | -16.5 | -3.5 |
| ST2G | R | -6.5 | 24.5 | -2.5 |
| ST2S | L | -2.5 | -25.5 | 0.5 |
| ST2G/ST2S | R | -3 | 22 | 0.5 |
| TEa | R | -8 | 25.5 | -8.5 |
| TEOM | R | 0.5 | 23.5 | -19 |
|  | R | 2 | 23.5 | -18 |
| TPO | L | 0.5 | -24 | -13.5 |
|  | R | 0.5 | 27 | -13 |
|  | R | -11 | 22.5 | -2 |
| Occipital Cortex |  |  |  |  |
| ProST | L | 0.5 | -13 | -20.5 |
| MT | R | 1 | 17 | -21.5 |
| V2 | R | -2.5 | 18.5 | -33 |
| V3 | R | 5.5 | 14 | -30.5 |
| V3V | R | 1 | 22 | -26.5 |
| V4/V5 | R | 17 | 18 | -26.5 |
| Basal Ganglia |  |  |  |  |
| Caudate | L | 0.5 | -7 | -3.5 |
| Putamen | R | -3.5 | 12.5 | 1 |
|  | L | -3.5 | -11.5 | 2.5 |
|  | L | -3.5 | -9 | 4.5 |
| SN | R | -7.5 | 5.5 | -6 |
|  | L | -7.5 | -3 | -10.5 |
| Cerebellum |  |  |  |  |
| Cb4 | L | -2.5 | -1.5 | -20 |
| Cerebellar Cortex | R | -7.5 | 8.5 | -20.5 |
| Cerebro-cerebellum | R | -4 | 4.5 | -29.5 |
|  | L | -8.5 | -8.5 | -28 |
|  | R | -6.5 | 2 | -20 |
|  | R | -7 | 1.5 | -19.5 |
| cp | L | -3.5 | -10.5 | -10 |
| Thalamus |  |  |  |  |
| CMn | L | -2.5 | -3 | -9 |
| LGN | R | -4 | 10 | -12.5 |
|  | L | -6 | -8.5 | -11.5 |
| MG | R | -5 | 5 | -12.5 |
| Pulvinar | R | 0.5 | 8.5 | -15.5 |
| VA | R | 0 | 0.5 | -2 |
| VPL | R | -2.5 | 8.5 | -7.5 |
|  |  |  |  |  |
| Hippocampus | L | -5.5 | -15 | -14.5 |
|  | L | -6 | -8.5 | -13 |
|  | R | -6.5 | 14.5 | -12 |
| Hypothalamus | R | -7.5 | 9 | -4 |
|  | L | -9 | -6 | -3.5 |
| Brainstem |  |  |  |  |
| Abducens Nucleus | R | -10.5 | 2 | -18.5 |
| IC | R | -2 | 0.5 | -16 |
| Oculomotor Nucleus | R | -6.5 | 3.5 | -10 |
| PPRF | L | -11 | -0.5 | -10.5 |
| SC | L | -5.5 | -3 | -14 |

Significant regions at a voxel level of p<0.001 corrected for multiple comparisons. Coordinates (mm) are given in monkey bicommissural space. Naming of BOLD-modulated regions was based on The Rhesus Monkey Brain atlas (Paxinos et al., 2000). ACC, anterior cingulate; AKL, auditory koniocortex, lateral part; Cb4, cerebellar lobule 4; CMn, centromedian thalamic nuclei; cp, cerebral puduncle, basal part; FEF, frontal eye field; IC, inferior colliculus; LGN, lateral geniculate nucleus; LIP, lateral intraparietal area; M1, primary motor cortex; MG, medial geniculate nucleus; MIP, medial intraparietal area; MT(V5) middle temporal area; PaI, parainsular area; PE, parietal area PE; PEC, parietal area PE, caudal part; PECg, parietal area PE, cingulate part; PFC, prefrontal cortex; PGa, PG associated area of the superior temporal sulcs; PGM, parietal area PG,medial part; PM, premotor area; PPRF, paramedian pontine reticular formation; ProST, prostriate area; S1, primary somatosensory cortex; S2, secondary somatosensory cortex; S2E, secondary somatosensory cortex, external part; SC, superior colliculus; SEF supplementray eye field; SN, substantia nigra; ST2G, superior temporal sulcus area 2, sulcal part; ST2S, superior temporal sulcus area 2, gyral part; TEa, temporal area TEa; TEOM, temporal area TE, occipitomedial part; TPO, temporal parietooccipital associated area in sts; V2, visual area 2; V3, visual area 3; V3V, visual area 3, ventral part; V4, visual area 4; VA, ventral anterior thalamic nucleus; VPL, ventral posterolateral thalamic nucleus; L, left; R, right.
