# Supplementary material for: Connectivity of the Primate Superior Colliculus Mapped by Concurrent Microstimulation and Event-Related fMRI
Source: PLoS One. 2008 Dec 11;3(12):e3928. doi: 10.1371/journal.pone.0003928 (PMC2592545; doi:10.1371/journal.pone.0003928)
Supplement: Table S2 — (0.13 MB DOC) [file pone.0003928.s003.doc]

Table S2. BOLD-modulated brain areas in monkey m2

|  |  |  |  |  |
| --- | --- | --- | --- | --- |
|  | Hemisphere |  | Coordinates |  |
|  |  | D-V | M-L | A-P |
| Frontal Cortex |  |  |  |  |
| ACC | R | 13 | 3.5 | 6.5 |
|  | R | 9 | 2 | 11 |
| ACC area 23c/24 | L | 13 | -2.5 | 0 |
| Area 13 | R | 4 | 5 | 11 |
| Principle sulcus, caudal pole | R | 12.5 | 16.5 | 5 |
| FEF | R | 14.5 | 10.5 | -1 |
|  | R | 14 | 10.5 | 4.5 |
|  | L | 13 | -8.5 | 8.5 |
| M1 | L | 22 | -10 | -6.5 |
|  | R | 20 | 5.5 | -4.5 |
| PM | R | 10.5 | 24 | -4.5 |
| Parietal Cortex |  |  |  |  |
| ACC area 23a | R | 5.5 | 7.5 | -19 |
| LIP | R | 13.5 | 7.5 | -24 |
| LIP/MIP | L | 15.5 | -20 | -14 |
| PECG | L | 16.5 | -7 | -22 |
| PG | L | 14 | -18 | -24 |
| PGM | L | 13 | -3 | -24.5 |
| PPt | R | 13 | 17 | -24.5 |
| S1/S2 | L | 21.5 | -8 | -13.5 |
| S2 | L | 7.5 | -15.5 | -10.5 |
| VIP | R | 6 | 12 | -24.5 |
| Temporal Cortex |  |  |  |  |
| AKL | L | 4.5 | -25 | -12 |
| FST | R | 3 | 21.5 | -24.5 |
|  | R | -5.5 | 19 | -12 |
| TFO | R | -5 | 24 | -22 |
| Occipital Cortex |  |  |  |  |
| MT/MST | R | 7 | 16.5 | -21 |
| V1 | R | 6 | 11.5 | -38 |
|  | R | -0.5 | 10 | -37 |
|  | L | 6 | -7.5 | -35.5 |
|  | R | -2.5 | 9 | -32 |
|  | L | 4 | -13.5 | -30.5 |
|  | L | -3 | -18 | -29.5 |
| V2 | R | -24.5 | 12.5 | -32 |
|  | L | 8.5 | -17.5 | -32 |
|  | L | 3 | -19 | -30.5 |
| V3 | R | 6 | 10 | -28.5 |
|  | R | 15 | 12 | -26.5 |
| Basal Ganglia |  |  |  |  |
| Caudate | R | 9 | 4.5 | -3.5 |
|  | L | 2 | -1 | 0 |
|  | L | 5.5 | -6.5 | 7.5 |
| SN | L | -7 | -8.5 | -9.5 |
|  | R | -10 | 5.5 | -8.5 |
|  |  |  |  |  |
| Cerebellum |  |  |  |  |
| Cb5 | R | -9.5 | 12.5 | -20 |
| Cerebro-cerebellum | R | -6 | 7 | -35.5 |
|  | R | -7.5 | 12.5 | -25.5 |
| Vermis | R | -3.5 | 4 | -24 |
|  | Mid | -7 | 0 | -24 |
|  |  |  |  |  |
| Thalamus |  |  |  |  |
| MD | R | 5 | 2 | -7.5 |
| Pulvinar | L | -2.5 | -10.5 | -19 |
|  | R | 2.5 | 10.5 | -9.5 |
| Pulvinar, lateral | L | 0 | -10 | -14 |
|  |  |  |  |  |
| Amygdala | L | -10 | -9 | 0.5 |
| Brainstem |  |  |  |  |
| IC | R | -6.5 | 4 | -19 |
| SC | L | -0.5 | -4.5 | -17.5 |

Significant regions at a voxel level of p<0.001 corrected for multiple comparisons. Coordinates (mm) are given in monkey bicommissural space. Naming of BOLD- modulated regions was based on The Rhesus Monkey Brain atlas (Paxinos et al., 2000). ACC, anterior cingulate; AKL, auditory koniocortex, lateral part; Cb5, cerebellar lobule 5; FEF, frontal eye field; FST, fundus of superior temporal sulcus; IC, inferior colliculus; LIP, lateral intraparietal area; M1, primary motor cortex; MD, medial dorsal thalamic nucleus; MIP, medial intraparietal area; MST, medial superior temporal area; MT(V5) middle temporal area; PECg, parietal area PE, cingulate part; PG, parietal area PG; PGM, parietal area PG,medial part; PM, premotor area; PPt, posterior parietal area; S1, primary somatosensory cortex; S2, secondary somatosensory cortex; SC, superior colliculus; SN, substantia nigra; TFO, temporal area TF, occipital part; V1, primary visual cortex; V2, visual area 2; V3, visual area 3; VIP, ventral intraparietal area; L, left; R, right.
